# Glycemic Index and Glycemic Load of Two Dishes Cooked with Alache (*Anoda cristata*) and Chaya (*Cnidoscolus aconitifolius*) Plants from the Traditional Mexican Diet

**DOI:** 10.1089/jmf.2022.0091

**Published:** 2023-06-13

**Authors:** Josefina Consuelo Morales-Guerrero, Reina Rosas-Romero, Ma. Amanda Mariscal-Gálvez, Fabiola Ayala-Alcántara, Héctor Bourges-Rodríguez

**Affiliations:** ^1^ Department of Food Science and Technology, Salvador Zubirán National Institute of Medical Sciences and Nutrition, Mexico City, Mexico.; ^2^ Faculty of Chemistry, National Autonomous University of Mexico, Mexico City, Mexico.

**Keywords:** alache, chaya, glycemic index, glycemic load

## Abstract

Correct nutrition is important for keeping good health; to attain that, the diet has to include vegetables such as quelites. The objective of this study was to determine the glycemic index (GI) and glycemic load (GL) of rice and a *tamal* prepared with and without two species of quelites: “alache” *(Anoda cristata)* and “chaya” *(Cnidoscolus aconitifolius)*. The GI was measured in 10 healthy subjects, 7 women and 3 men, with the following mean metrics: age, 23 years old; body weight, 61.3 kg; height, 1.65 m; body mass index, 22.7 kg/m^2^; and basal glycemia, 77.4 mg/dL. Capillary blood samples were collected within 2 h after the meal. White rice (rice with no quelites) had a GI of 75.35 ± 15.6 and a GL of 36.17 ± 7.8; rice with alache had a GI of 33.74 ± 5.85 and a GL 33.74 ± 1.85. White tamal had a GI of 57.33 ± 10.23 and a GC of 26.65 ± 5.12; tamal with chaya had a GI of 46.73 ± 22.1 and a GL of 23.36 ± 11. The GI and GL values recorded for the combination of quelites with rice and tamal confirmed that quelites could be a good alternative for healthy diets.

## Introduction

Mexico is a megadiverse country that harbors 10–12% of the planetary species and one of the countries with the greatest biological biodiversity. Of the Mesoamerican species domesticated in the country, 142 are grown in plots smaller than five hectares.

In Mexico, a large number of domesticated species have limited use due to social, cultural, and geographic factors. Neglected and underutilized species (NUS)^1^ are defined as species or varieties of traditional/ancestral use adapted to specific agroecological niches of high nutritional value but with reduced or decreasing cultivation and use. On top of it, many NUS are endangered of extinction due to changes in consumption and production patterns associated with lower demand, agroecological changes, and loss of traditional knowledge related to their growth and cultivation. Besides, scarce information is available regarding local cultivation, post-harvest, and processing practices of NUS.^1^

Poverty and marginalization are one of the faces of the food production model in the rural sector, especially in rain-fed regions where population segments suffering from undernutrition are common. The other face is malnutrition: obesity affects almost one third of the adult and children population in the country,^2^ considered the highest rate worldwide, only after the United States of America, according to the data published by the National Survey on Health and Nutrition 2018.^3^ Some population segments rank first worldwide, as indicated by the index of child obesity.

These tendencies are closely related to the eating habits in Mexico, which seem to have changed after the opening of agricultural produce to international markets, and that partly explain the adverse effects observed by the increased incidence of diabetes mellitus and obesity in the Mexican population.^4^

Consumption of wild plants collected by humans goes back to prehistoric times and is still prevalent today. The traditional use of these resources is closely related to ethnic and indigenous groups that inhabit different environments and create a wide range of knowledge, including handling and cooking techniques that have significantly broadened the options for using traditional plants as food, as described by Casas and Caballero^5^ and Mera et al.^6^

Since the times of the Spanish conquest, many chroniclers, naturalists, anthropologists, and ethnobiologists have documented, to a greater or lesser extent, the array of plants used as food. Many of these plants are considered quelites, that is, plants whose leaves and tender stalks are consumed as vegetables.

The knowledge and use of quelites in Mexico started to decline since the times of the Spanish conquest of Mexico because the conquerors did not accept the consumption of wild plants. It has been calculated that quelites use has declined by 55–90% over the past 500 years as a result of the negative effect of colonization on this valuable food resource.

The local use and knowledge of quelites still prevail in regions where indigenous people grow them to prepare traditional dishes.^7^

The listing of plants consumed today by the Mexican population indicates that only a small percentage of the plants recorded in pre-Columbian times are still in use. Some of these species have been excluded from commercial crops because they are only collected, not planted, or grow freely in family plots; these are considered weeds when found in commercial plantations. The growth and collection of quelites are not included in agricultural censuses, and, therefore, no records are kept on their economic, nutritional, and gastronomic contributions to the national systems, although they remain as a major nutritional asset in rural communities.

Quelites,^8,9^ which include both herbaceous and woody species, have a great potential for improving the Mexican diet and, therefore, the quality of life. Quelites are part of the pre-Hispanic agroecosystem based on maize, beans, and squash, named *milpa* in Spanish.^10^

The National Herbarium of the Institute of Biology at Universidad Nacional Autónoma de México (UNAM) safeguards an inventory of 244 species of quelites, including 121 genera in 46 botanical families, distributed throughout the national territory. A selection of the most common quelite species is listed in Table 1.^11^

**Table 1.** Selection of Well-Known Quelites Consumed as Food in Central Mexico

Common name	Scientific name
Alache	*Anoda cristata*
Atenquelites	*Phaseolus sp germinados*
Berros	*Nasturtium officinale*
Chaya	*Cnidoscolus aconitifolius*
Chepil (o chipilín)	*Crotalaria pumila*
Flores de calabaza	*Cucurbita pepo*
Flores de yuca	*Yucca sp.*
Guías de calabaza	*Cucurbita pepo*
Guías de chayote	*Sechium edule*
Hierbamora	*Solanum nigrescens*
Hoja Santa (o acuyo)	*Piper auritum*
Huauquelite	*Chenopodium berlandieri* subsp. *nuttalliae*
Huauzontle	*Chenopodium berlandieri* subsp. *nuttalliae*
Lengua de vaca	*Rumex mexicanum*
Pápalo quelite	*Porophyllum macrocephalum*
Pipicha	*Porophyllum linaria*
Quelite cenizo	*Chenopodium berlandieri*
Quelite de frijol	*Phaseolus* sp
Quintonil	*Amaranthus hybridus* and *A. hypochondriacus*
Romerito	*Suaeda edulis*
Verdolaga	*Portulaca oleracea*

Source: Adapted from Linares et al.^11^

**FIG. 1.**
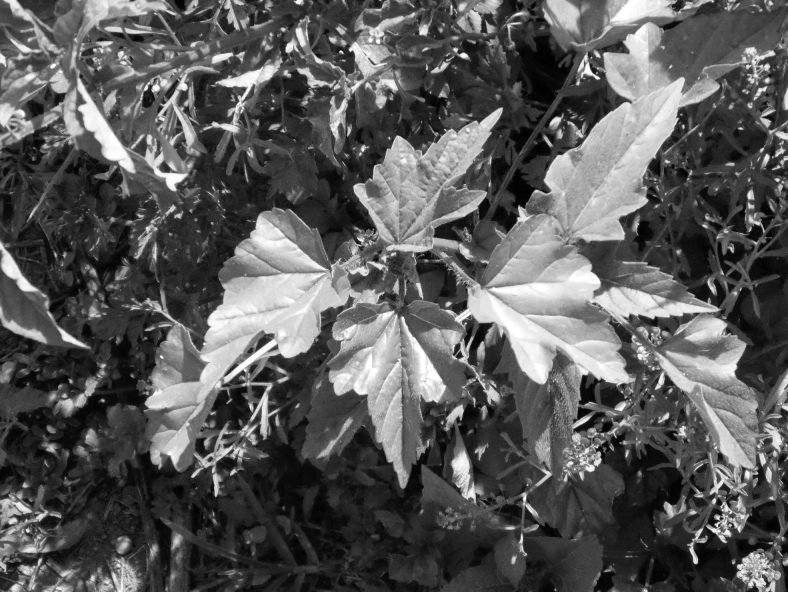
Alache plant. Photo courtesy of Dr. Edelmira Linares, Biology Institute, National Autonomous University of Mexico.

**FIG. 2.**
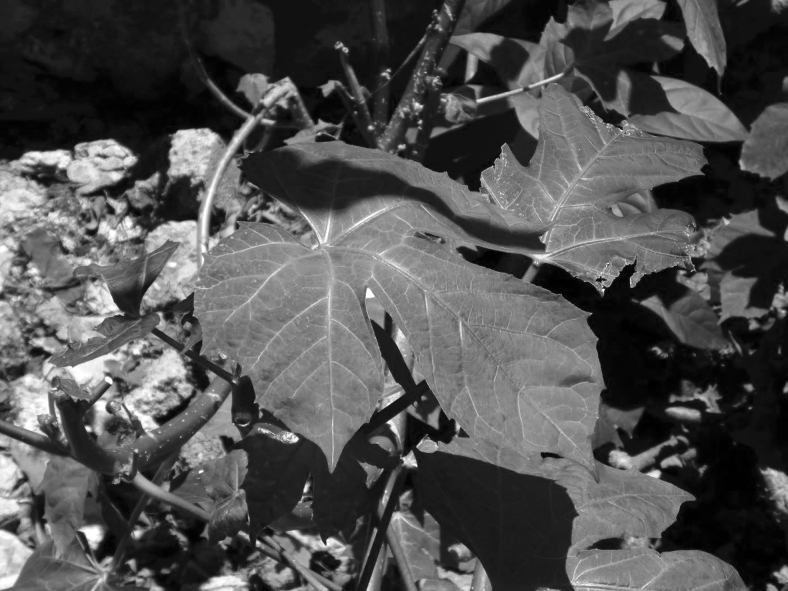
Chaya plant. Photo courtesy of Dr. Robert Bye, Biology Institute, National Autonomous University of Mexico.

Studies addressing these NUS in Mexico are currently required to expand our knowledge about their chemical composition and nutritional value, to utilize them as rich nutritional foods^12^ that can contribute to improving the Mexican diet. The main characteristics of alache (Fig. 1) and chaya (Fig. 2), two common quelite varieties, are described in Table 2.

**Table 2.** Name, Botanical Characteristics, and Uses as Food and Herbal Medicine^13^ of Quelites Analyzed: Alache and Chaya

Common name: Alache, spurred Anoda, crested Anoda, and violetta; its name in nahuatl, “alaztic,” means slippery				
Scientific name: Anoda cristata (L.) Schelcht (Fig. 1)				
Area of origin	Found in	Botanical description	Uses as food	Medicinal uses
Mexico and Central America	Most states of the Mexican Republic	Annual herb, 30–70 cm high, with purple flowers. It blooms from mid-September to late October. Peasants have allowed alaches to grow freely in maize plots for centuries.^14^	Leaves, flowers, and stems are eaten by indigenous peoples of Mexico as part of an important tradition. Alache is very much appreciated because of its flavor^15^	It has been traditionally used to alleviate fever and reduce flu symptoms and coughing. It is taken as an infusion, either alone or mixed with chamomile. Juarez et al.^16^ published that alache (*Anoda cristata*) extracts show hypoglycemic and anti-hyperglycemic effects

Common name: Chaya; in the Mayan language, chaycol and Chaya^17^				
Scientific name: Cnidoscolus aconitifolius (Fig. 2)				
Area of origin	Found in	Botanical description	Uses as food	Medicinal uses
Coastal regions of the Gulf of Mexico and the Caribbean	Indigenous peoples of Yucatan, Mexico	*Cnidoscolus aconitifolius* belongs to the family Euphorbiaceae, a shrub of southern Mexico that can grow up to 5–6 m high. It is a tropical plant adapted to grow in humid and dry environments, at altitudes from sea level to ∼1500 m above sea level. It is well known and cultivated in Mesoamerica^13^ (and appreciated due to its nutritional, medicinal, and ornamental values).	The nutritional value of chaya is based on its contents of calcium, potassium, iron, phosphorus, and some vitamins.^15^ Leaves are consumed in diverse culinary preparations that are traditional in southeast Mexico	Chaya is considered to have antioxidant properties due to its contents of vitamin A, C, and E and also contributes to reduce the severity of diabetes^18^ A chaya infusion may be recommended to reduce blood lipid levels

The present study assessed the glycemic index (GI) of two quelite species included as ingredients in two dishes: white rice and tamal. The term GI, proposed by Dr. Jenkins in 1981,^19^ is defined as the numerical expression describing the speed of carbohydrate absorption from a particular food. The GI classification has been globally adopted by organizations such as FAO and WHO (ISO26642, 2010).^20^

The GI is measured on a 0–100 scale, considering table sugar (sucrose) as having a value of 100. Carbohydrate-rich foods may have high GI values and readily increase blood glucose concentrations. GI values of 70 or higher are considered high GI values. Moderate GI values range from 56 to 69, and values lower than 55 are considered low GI values. Most vegetables are considered low GI values, with a few exceptions.

Carbohydrates are the primary drivers of increased blood glucose levels, although lipids and protein may also affect glycemic responses or contribute to maintaining glycemia. Therefore, each food has particular factors that condition the glycemic response, such as:

- Amylose/amylopectin ratio: Side chains in amylopectin and their water interactions make it more prone to enzymatic attack, leading to a faster and higher glycemic response.
- Degree of food processing: Highly processed foods elicit a higher glycemic response.
- Lipid content: Lipids produce complexes with carbohydrates, blocking the contact of the enzymes that break polysaccharide linkages with their substrate.
- Protein content: Proteins stimulate the secretion of gastric fluid, limiting the function of amylases. When carbohydrate-rich food is ingested, high protein and lipid contents slow down carbohydrate digestion and absorption, resulting in low GI food.^21^

Besides the characteristics of the food consumed, the physiological and health status of the human subject influences the glycemic response; all of the above determine the metabolic route, followed by nutrients in food and their effect on glycemia.^22^

Another key concept is glycemic load (GL), defined in 1997, which considers the effect on the blood glucose level of a person after having consumed food or a dish. It is based on the concept of GI, calculated by multiplying the GI of a food item by its carbohydrate content, divided by 100.

GI and GL are useful tools for the selection of food types that provide protection in case of chronic diseases, and both indices can be considered indicators of the nutritional quality of the diet. However, they should not be used in isolation, and their limitations need to be considered in both the clinic and public health.^23^

## Materials and Methods

This study was approved by the IRB Committee of the INCMNSZ (reference 2101).

### Selection of participants

Subjects interested in participating were invited via the internet, considering the following inclusion criteria: (1) the subject does not suffer a chronic degenerative disease, (2) the subject is not undergoing pharmacological or hormonal treatment, and (3) the subject has a body mass index (BMI) lower than 25 kg/m^2^.

After suitable study subjects were selected, each was interviewed by the site (INCMNSZ) nutritionist, who recorded size, height, weight, and calculated the BMI. The diet consumed before the study should not include legumes to avoid biasing the results as legume species decrease the GI. Participants were instructed to refrain from consuming alcoholic beverages, smoking, or receiving any hormonal treatment over the course of the study.

### Blood samples

Participants attended a study site appointment after a 12 h fasting. At time 0, a baseline sample of capillary blood was collected with an automated lancet puncture on a fingertip after the oral administration of a 250 mL solution containing 25 g anhydrous glucose; subsequently, samples were collected at 15, 30, 45, 60, 90, and 120 min post-glucose administration. Blood samples were collected in a capillary tube with heparin, then transferred to a 0.5 mL Eppendorf tube, and tested for glucose concentration with a Biochemistry Analyzer YSI 2700 according to the ISO Standard 26642.^20^ A glucose reference was tested in triplicate as a control for the following food assays: white rice (cooked without *alache*) and rice cooked with *alache*, as well as white tamal (cooked without *chaya*) and tamal cooked with *chaya*. Data were plotted, and the area under the curve (AUC) was calculated using the trapezoid method.

### GI and GL

GI is the ratio comparing the rise in blood glucose concentration caused by the test food to the one observed when consuming a reference food (in this case, glucose solution) 2 h after ingestion, calculated according to the following equation

$$GI=\left[testfoodAUC∕glucosestandardAUC\right]\times 100$$

where AUC is calculated with the trapezoid method, and glucose standard = 25 g anhydrous glucose in 250 mL solution.

Glucose load is the amount of carbohydrates in the test food serving, calculated according to the following equation:

$$GL=\left(GIoffood\times gofcarbohydratefoodperserving\right)∕100$$

GL values are considered to be high when the result exceeds 20, whereas food with a low GL yields values lower than 10.^24^

### Test food preparation

#### White rice and rice with alaches

Precooked rice was purchased in a supermarket and cooked following the label instructions. Rice with alaches (test food) was prepared according to the recipe published by Linares et al.,^11^ using 200 g of alaches in broth mixed with 100 g of precooked rice and boiled until the mixture was well cooked. Separate 193 g portions of rice, each containing 25 g of carbohydrates, were frozen at −18°C until used; 250 g portions of rice with alaches were also kept frozen until used.

#### White tamal and tamal with chaya

White tamales were cooked according to Gálvez.^25^ For tamales with chaya, 100 g portions of raw cornmeal dough were mixed with 15 g of chopped raw chaya leaves, then wrapped individually with banana leaves, and steam cooked for 45 min.

## Results

The profile of study participants in terms of age, sex, height, and BMI is shown in Table 3, and the results are summarized in Table 4. These indicate the benefits associated with the consumption of rice with alaches, which led to lower GI values relative to the reference food serving. According to the results obtained in this study, white rice and rice with alaches are classified as having high and intermediate GI, respectively. For its part, white tamal classifies as intermediate GI, whereas tamal with chaya results in a low GI.

**Table 3.** Characteristics of the Healthy Volunteers Participating in the Study

Parameter	Total (*n* = 10)	Females (*n* = 7)	Males (*n* = 3)
Age (years)	23.1 ± 1.52	23 ± 1.41	23.3 ± 2.08
Weight (kg)	61.03 ± 7.7	56.8 ± 3.7	71 ± 3.6
Height (m)	1.65 ± 0.09	1.6 ± 0.06	1.75 ± 0.03
BMI (kg/m^2^)	22.7 ± 1.30	22.5 ± 1.4	23.1 ± 1.03

BMI, body mass index.

**Table 4.** Glycemic Index Values for Rice, Rice with Alaches, Tamales, and Tamales with Chaya, According to the Results for Each Participant

No. of participant	GI of white rice	GI of rice with alaches	GI of tamales	GI of tamales with chaya
1	87.8	68	48.9	20.6
2	94.8	57.5	70.8	41
3	76.9	76.5	49.4	64
4	73.7	85.2	64.4	80
5	72.8	58.8	53.4	67.7
6	51	68.7	41.6	21.6
7	67.6	44.8	49.3	38
8	43.9	70.3	69.7	68.5
9	71	79.3	58.8	44.9
10	84	65.9	67	21
Mean ± SD	72.4 ± 15.6	67.50 ± 11.72	57.33 ± 10.23	46.73 ± 22.13

GI, glycemic index; SD, standard deviation.

## Discussion

Mexico is a major exporter of agroindustrial products, ranking 12th in food production worldwide and 11th in the list of top world producers of agricultural produce, so Mexican foods are well positioned in the global market. Despite it all, the segments of the Mexican population with the lowest income, living in rural areas of the country, are underprivileged groups suffering food insecurity; however, the large variety of native plant species, including quelites, can positively impact the diet of the low-income segments.

Quelites, that is, Mexican native wild plant species that have been consumed since pre-Hispanic times, are central elements that supplement the diet of the rural population in Mexico, sometimes even being the main course in traditional diets. More than 350 species are considered quelites, including plant species in several botanical families. Examples are *Amaranthus* spp. (*quintonil*), *Chenopodium berlandieri* (*quelite cenizo*), and *Portulaca oleracea* (*verdolaga*),^26^ which constitute the most popular species of quelites used in the Mexican cuisine in dishes such as tamales, soups, quesadillas, and salads. These species provide fragrance, color, and varied flavors to food, supplementing it with dietary fiber, inorganic nutrients (Ca, K, Mg, Mn, Zn, and P), vitamins A and C, and bioactive compounds such as polyphenols and betalains, among other nutraceuticals that confer a high antioxidant capacity to these plants.

Quelite species are usually collected during the rainy season, when they are available in regional markets and in some supermarkets, being sold at low prices. Despite being affordable and important food ingredients, quelite consumption has declined due to the current trend toward lower demand by consumers. Besides, quelites are being eliminated from crops by chemical herbicides applied to crop fields before planting instead of hiring laborers for this task, and because of the rising temperature and more frequent and intense droughts. However, the primary driver of this trend is the huge shift in the preference of consumers, who now eat mainly processed foods, frequently formulated with very high energy content and added sugars, and almost devoid of natural vitamins, minerals, and phytochemicals. This change has led to a higher incidence of chronic degenerative diseases, such as type 2 diabetes mellitus and cardiovascular disease.^27^

A dietary plan including low-GI foods^28^ benefits the health status and quality of life of consumers by improving glucose tolerance, producing satiety for longer times, and strengthening the immune system. To achieve this goal, low-GI foods have to be consumed because they provide a stable energy supply to the body in addition to providing antioxidants and essential nutrients that slow down aging.^29^ GI and GL are valuable tools for the selection of foods that protect against chronic diseases.^30^

The importance of the GI lies in the benefits observed in studies addressing diet planning for diabetic patients and individuals requiring treatment for obesity, overweight, or cardiovascular disease. Besides, GI is now being applied as part of the recommendations for planning healthy diets because of the effects of low-GI food consumption in the prevention of the health conditions just mentioned.^31^

Natural yogurt, whole-wheat pasta, lentils, prunes, grapefruits, peaches, pears, tomato, and green vegetables, among many others, produce lower glycemic variability, which benefits both healthy individuals and patients suffering from type 2 diabetes mellitus.

In conclusions, mixing alaches or chaya with high-carbohydrate foods can lead to a lower GI. It is important to remember, however, that these dishes have a high GL; therefore, they have to be consumed in moderation. The ultimate recommendation is that, in general, high-carbohydrate dishes should be consumed together with large portions of vegetables. The results of the present study indicate that the two quelite species studied have an interesting potential and should be repositioned as foods commonly used in Mexico, a country where a high incidence of diabetes mellitus currently prevails.
